# Application of Natural Antioxidants as Feed Additives in Aquaculture: A Review

**DOI:** 10.3390/biology14010087

**Published:** 2025-01-17

**Authors:** Xiaodan Hu, Wenjing Ma, Disen Zhang, Zikun Tian, Yuanqiang Yang, Yi Huang, Yuhang Hong

**Affiliations:** 1 Key Laboratory of Application of Ecology and Environmental Protection in Plateau Wetland of Sichuan, Xichang University, Xichang 415000, China; 2 Key Laboratory of Animal Disease Detection and Prevention in Panxi District, Xichang University, Xichang 415000, China

**Keywords:** natural antioxidants, plants, aquaculture, feed additives, growth, anti-stress

## Abstract

Oxidative stress, a critical factor affecting the health and productivity of aquatic organisms, arises from the imbalance between reactive oxygen species (ROS) production and antioxidant defenses. In aquaculture, natural antioxidants have gained increasing attention as sustainable feed additives to mitigate oxidative damage, enhance immune responses, and improve overall growth performance. This review provides a comprehensive synthesis of the antioxidative mechanisms of key natural antioxidants, including carotenoids, polysaccharides, vitamins, polyphenols, and flavonoids. By neutralizing ROS and modulating cellular signaling pathways such as Nrf2/ARE, these compounds offer significant protective effects against oxidative damage in aquatic species. The manuscript consolidates recent advancements in antioxidant research, highlighting their practical applications in feed formulation and their role in promoting sustainability in aquaculture. This review aims to provide an integrative framework for understanding natural antioxidants’ potential, guiding future research and practical implementation in aquaculture systems.

## Simple Summary

Oxidative stress is a significant challenge in aquaculture, often triggered by environmental factors such as poor water quality, high stocking densities, temperature fluctuations, and exposure to contaminants like ammonia and heavy metals. These conditions lead to the excessive production of harmful molecules, known as reactive oxygen species (ROS), which can overwhelm the body’s natural defenses and cause cellular damage. Natural antioxidants, found in plants and other natural sources, have the potential to reduce oxidative stress by neutralizing ROS and protecting aquatic species. This review explores the role of various natural antioxidants, including carotenoids, polysaccharides, vitamins, polyphenols, and flavonoids, in promoting better health and resilience in fish and other aquatic animals. These antioxidants not only improve immune function and stress tolerance but also enhance growth and product quality. The findings provide insights into how natural antioxidants can serve as eco-friendly alternatives to synthetic additives, supporting sustainable aquaculture practices. By understanding their mechanisms and benefits, this study offers guidance for researchers and farmers to improve aquaculture systems, ensuring healthier aquatic animals and more sustainable production for the growing global demand for seafood.

## 1. Introduction

Aquaculture, as a vital component of modern agriculture, not only serves as an essential carrier for aquatic ecosystems but also has a profound impact on global food supply. However, oxidative stress has emerged as a critical factor limiting animal health and growth performance in aquaculture. Oxidative stress affects the physiological metabolism of aquatic animals and weakens their immune systems, thereby reducing farming efficiency and increasing the risk of diseases [1]. It adversely influences animal physiological health and production performance, ultimately affecting the economic efficiency of the aquaculture industry [2]. In addition, anthropogenic noise is one of the triggers for the evolution and changes of antioxidative system in various aquatic organisms [3,4,5]. In nearshore aquaculture systems, the noise from fishing boats and maritime transportation vehicles, as well as objects such as aerators and vehicles that can produce noise in terrestrial aquaculture systems, have been studied to suggest that these noises may to some extent affect oxidative status and immune parameters [6]. The use of antioxidants to mitigate oxidative stress in aquaculture is thus of great significance. At present, most feed additives with antioxidative properties are applied primarily in livestock and poultry farming [7], with relatively late research and development in aquaculture applications. Commercially available antioxidants are generally classified into two categories: natural antioxidants derived from plants, such as astaxanthin, resveratrol, plant polysaccharides, vitamins, and flavonoids [8], and synthetic antioxidants, including butylated hydroxyanisole (BHA) and butylated hydroxytoluene (BHT).

Natural antioxidants, being naturally ingested by humans, are considered less harmful, safer, and more acceptable to consumers compared to synthetic counterparts [9]. They are widely sourced from many common vegetables, such as spinach, carrots, and tomatoes. This paper reviews existing studies on the application of natural antioxidants in aquaculture feed, aiming to facilitate further development of natural antioxidants and promote the growth of the aquaculture industry.

## 2. Oxidative Stress

Oxidative stress refers to an imbalance between oxidation and antioxidation within the body, resulting in the excessive production of reactive oxygen species (ROS) and reactive nitrogen species (RNS). ROS include oxygen free radicals such as superoxide anion (O_2_^−^) and hydroxyl radical (·OH), as well as non-radical species like hydrogen peroxide (H_2_O_2_). At low concentrations, ROS have physiological roles, such as aiding immune cells in eliminating foreign pathogens and functioning as signaling molecules in cellular communication.

However, due to their high chemical reactivity, excessive ROS can cause oxidative damage to biological macromolecules such as DNA, proteins, and lipids. This damage is associated with the development of aging, cancer, and various other diseases. ROS can also induce inflammatory infiltration of neutrophils, increase protease secretion, and generate large quantities of oxidative intermediates, ultimately leading to tissue and cellular damage [10]. In aquaculture, oxidative stress is particularly detrimental due to its impacts on both physiological and economic aspects. For instance, lipid peroxidation caused by ROS can compromise the nutritional quality of fish fillets, reducing their market value [11]. Moreover, oxidative stress can suppress immune function, making fish more susceptible to diseases and increasing mortality rates, which pose challenges to sustainable production [12].

### 2.1. Oxidative System

The oxidative mechanism of oxidative stress involves the combined effects of endogenous factors, such as mitochondrial aerobic respiration; arachidonic acid metabolism; enzymatic pathways like xanthine oxidase; and exogenous factors, such as radiation, ultraviolet light, and harmful chemicals. These factors lead to the production of highly reactive molecules within cells, including ROS and RNS. When these molecules are not promptly eliminated and accumulate excessively, they can cause adverse effects such as protein denaturation, DNA damage, and lipid peroxidation. These effects disrupt cellular functions, induce apoptosis, and may ultimately lead to the development of diseases [13].

During key metabolic processes, such as glycolysis, the tricarboxylic acid (TCA) cycle, and β-oxidation, electron-rich reducing cofactors participate in oxidation reactions to release energy. However, in the mitochondrial respiratory chain, approximately 2–3% of electrons may leak and react incompletely with oxygen molecules, generating O_2_^−^. Superoxide dismutase (SOD) within mitochondria converts O_2_^−^ into H_2_O_2_, which diffuses into the cytoplasm. There, in the presence of iron ions, H_2_O_2_ undergoes a Fenton reaction, producing highly reactive hydroxyl radicals that cause further oxidative damage to cells. Additionally, O_2_^−^ may contribute to the formation of other toxic substances, exacerbating cellular damage [14].

**Figure 1:**
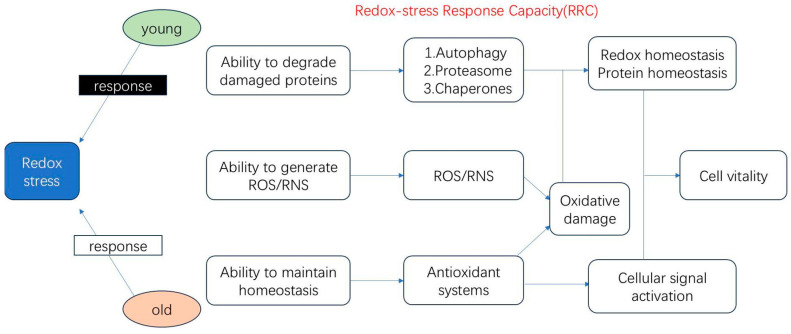
Schematic diagram of the role of antioxidative response in aging.

The oxidative stress response is closely linked to aging. As individuals age, the body’s antioxidative capacity declines, reducing its efficiency in clearing oxidative agents. This decline leads to the accumulation of free radicals in cells and tissues, impairing physiological functions, accelerating the aging process, and significantly increasing the risk of chronic diseases (Figure 1).

### 2.2. Antioxidative System

The natural regulation of ROS in animals is primarily managed by enzymatic antioxidative systems, non-enzymatic antioxidative systems, and antioxidative-related proteins. These systems work synergistically in aquatic animals to finely regulate and maintain the oxidative–antioxidative balance and homeostasis, ensuring healthy growth and sustainable productivity [15].

The enzymatic antioxidative system consists mainly of SOD, catalase (CAT), and glutathione peroxidase (GSH-Px). These enzymes protect against oxidative damage by breaking down reactive oxidative molecules [16]. The non-enzymatic antioxidative system includes small molecules such as vitamin C (Vc), vitamin E (Ve), glutathione, flavonoids, ubiquinone, and carotenoids, which neutralize or scavenge free radicals to mitigate oxidative stress [17]. Additionally, antioxidative proteins such as metallothionein and ferritin play vital roles in the oxidative stress response [18].

Multiple cellular signaling pathways are closely associated with the oxidative stress response, including the Nrf2-Keap1 pathway [19], MAPK/NF-κB pathway [20], and PI3K-Akt pathway [21]. In zebrafish, the Nrf2-Keap1 pathway significantly regulates the expression of antioxidative enzyme genes [22]. Under normal physiological conditions, Nrf2 is bound to the Keap1 protein in the cytoplasm, with a small portion translocating to the nucleus to maintain basal expression of antioxidative genes. When free radical levels rise, causing redox imbalance, Nrf2 dissociates from the Keap1-Nrf2 complex, translocates to the nucleus in large quantities, and binds to antioxidant response elements (AREs), promoting the expression of antioxidative enzymes and restoring cellular homeostasis [23]. Among enzymatic antioxidative systems, SOD serves as the first line of defense against oxygen free radicals. It efficiently catalyzes the dismutation of superoxide anions into hydrogen peroxide and oxygen (2O_2_^−^ + 2H^+^ → H_2_O_2_ + O_2_). Subsequently, CAT, GSH-Px, and peroxiredoxins (PRX) convert H_2_O_2_ into water and oxygen, completely neutralizing reactive free radicals to protect the body from damage [17]. Some researchers suggest that CAT assists GSH-Px in antioxidative reactions [24], effectively converting H_2_O_2_ into harmless water and oxygen [25].

The non-enzymatic antioxidative system involves antioxidants like vitamins A, C, and E; glutathione; melatonin; uric acid; and bilirubin. These non-enzymatic antioxidants eliminate excess free radicals or reduce their activity, interrupting free radical chain reactions to mitigate oxidative stress and protect cells from damage [26]. Studies have shown that vitamin C, as a potent antioxidant, alleviates oxidative stress in fish. For instance, in grass carp kidney cells exposed to cadmium (Cd)-induced oxidative stress, vitamin C supplementation enhanced the activities of SOD, GSH, and CAT [27].

In summary, the collaboration between enzymatic and non-enzymatic antioxidative systems is crucial for maintaining the oxidative–antioxidative balance in aquatic animals. Further research into these mechanisms provides a theoretical basis for developing antioxidative strategies to improve aquatic animal health and production performance.

### 2.3. Effects of Oxidative Stress on Aquatic Organisms

#### 2.3.1. Major Causes of Oxidative Stress in Aquatic Organisms

(1) Environmental Pollution:

Environmental pollution is a significant factor inducing oxidative stress in aquatic animals. Toxic substances, including heavy metals such as mercury, lead, and cadmium, as well as pesticides and industrial wastewater, can directly or indirectly increase the production of reactive oxygen species. These pollutants interact with the redox systems within cells, leading to excessive free radical accumulation, triggering oxidative stress responses, and ultimately causing damage to cellular functions and tissue health [28].

(2) Climate Change:

Climate change, particularly global warming and ocean acidification, poses severe threats to the health of aquatic organisms. Rising water temperatures and reduced dissolved oxygen levels due to global warming disrupt the physiological balance of aquatic animals. Ocean acidification, which results in a decrease in pH, further compromises the acid–base balance and the skeletal formation of calcifying organisms. The combined effects of reduced oxygen transport capacity and acid–base imbalance heighten the risk of oxidative stress, negatively impacting growth, reproduction, and immune functions of aquatic organisms [29].

(3) Changes in Water Quality:

Water quality is a critical determinant of the health and productivity of aquatic organisms in aquaculture systems. Parameters such as dissolved oxygen (DO), temperature, pH, ammonia levels, nitrite concentrations, and organic matter content significantly influence the oxidative balance within aquatic species. Deviations in these parameters can lead to elevated production of reactive oxygen species, thereby inducing oxidative stress.

Dissolved Oxygen (DO): Low oxygen levels (hypoxia) are a common stressor in intensive aquaculture systems. Hypoxic conditions disrupt mitochondrial function, leading to excessive ROS production in fish [30].

Ammonia and Nitrite Toxicity: Accumulation of nitrogenous wastes, such as ammonia (NH_3_) and nitrites (NO_2_^−^), is a frequent issue in aquaculture, particularly in systems with insufficient water exchange or poor biofiltration [31]. Ammonia toxicity induces oxidative stress by disrupting ion balance and increasing ROS generation, leading to lipid peroxidation and tissue damage in fish and other aquatic species [32].

Temperature Fluctuations: Sudden changes in water temperature can stress aquatic species by altering metabolic rates and oxygen demand. Elevated temperatures are particularly problematic as they accelerate ROS production through enhanced metabolic activity, while simultaneously reducing the solubility of oxygen in water, compounding oxidative damage [33].

pH Variations: Extreme pH levels, either acidic or alkaline, can destabilize cellular homeostasis in aquatic organisms. Acidic conditions, for instance, increase the permeability of gill membranes to toxic metals such as aluminum, which catalyze ROS formation, thereby exacerbating oxidative stress [34].

Organic Matter and Eutrophication: The accumulation of organic waste from uneaten feed and feces promotes microbial activity, consuming oxygen and releasing toxic byproducts such as hydrogen sulfide (H_2_S). Hydrogen sulfide exposure has been shown to induce severe oxidative stress by inhibiting mitochondrial respiration in aquatic organisms [35].

Pollutants and Xenobiotics: Aquaculture environments are often contaminated with pesticides, heavy metals, and other xenobiotics, which act as external ROS generators. For instance, copper exposure in water bodies triggers oxidative stress by interfering with antioxidant defense systems [36].

#### 2.3.2. Harmful Effects of Oxidative Stress on Aquatic Organisms

(1) Cellular and Tissue Damage:

Oxidative stress can cause protein denaturation, lipid peroxidation, and apoptosis in aquatic organisms, ultimately leading to cellular damage [37]. For instance, after 48 h of exposure to high copper levels, the expression of *CAT* mRNA in the liver increases by 2.5 times. Moreover, as exposure duration extends, the expression of *COX-17* mRNA in the liver also shows a gradual increase, reaching an eightfold rise after 48 h. These changes exacerbate oxidative damage and alter the antioxidant capacity of cells [38].

(2) Fluctuations in Antioxidant Enzyme Activity:

Oxidative stress affects the antioxidative enzyme systems of aquatic organisms, including key enzymes like SOD, CAT, and GST. Under oxidative stress, the activities of these enzymes may either increase or decrease, impacting the organism’s overall antioxidative capacity [39].

(3) Impairment of Physiological Functions:

Oxidative stress adversely affects the physiological functions of aquatic organisms by disrupting energy metabolism and ATP production [40]. It can also reduce growth performance. For example, red sea bass consuming oxidized fish oil without vitamin C supplementation exhibit significantly lower growth performance [41].

(4) Negative Effects of Environmental Pollutants:

Pollutants such as heavy metals (e.g., mercury, lead, cadmium) [42] and other chemicals (e.g., polycyclic aromatic hydrocarbons (PAHs), pesticides) [43] can enhance ROS production and disrupt antioxidative defense systems, triggering oxidative stress responses in fish.

In summary, oxidative stress negatively impacts fish growth and development in aquaculture, driving an increasing demand for antioxidants. However, growing consumer concerns about food safety and health have led to restrictions and controversies surrounding synthetic antioxidants. In contrast, natural antioxidant extracts and compounds, being naturally present in food and posing lower health risks, are gaining popularity. These natural antioxidants not only effectively inhibit lipid oxidation but also offer additional health benefits, such as antibacterial properties [44]. Against this backdrop, this study aims to delve into the current applications of natural antioxidants as feed additives in aquaculture and their potential roles in improving fish health, enhancing immune functions, and stabilizing feed quality.

## 3. Natural Antioxidants

### 3.1. Carotenoids: Mechanisms, Applications, and Aquaculture Relevance

Carotenoids are a family of over 600 lipophilic plant pigments that serve as the basis for the coloration of many organisms in nature [45]. Common carotenoids include beta carotene, lycopene, lutein, astaxanthin, and zeaxanthin. The following table shows common carotenoids and their extraction techniques and sources (Table 1).

The “core” structural element of carotenoids is a polyene skeleton composed of a series of conjugated C=C bonds; this special characteristic is mainly responsible for the ability of free radicals to interact with singlet oxygen, making it an effective antioxidant [46]. Carotenoids may help prevent lipid peroxidation, reduce cellular oxidative stress, and decrease inflammatory responses in tissues [47]. In 1957, Fujimori and Livingston demonstrated that carotenoids have the ability to inhibit the triplet state of chlorophyll (3chl) in vitro [48]. Under photochemical induced oxidation, carotenoids have the ability to quench the first potential harmful intermediate 3S (triplet sensitizer) at a very significant rate. Taking chlorophyll as an example, 90% of Chi will be extinguished by carotenoids, leading to the inhibition of any potential reactions [49]. They have garnered significant attention due to their remarkable antioxidant activity [50]. Among them, astaxanthin, a representative carotenoid, stands out for its exceptional antioxidative properties, efficiently scavenging oxidative agents in the body and emerging as a highly promising natural antioxidant [51].

#### 3.1.1. Sources and Extraction of Astaxanthin

**Figure 2:**
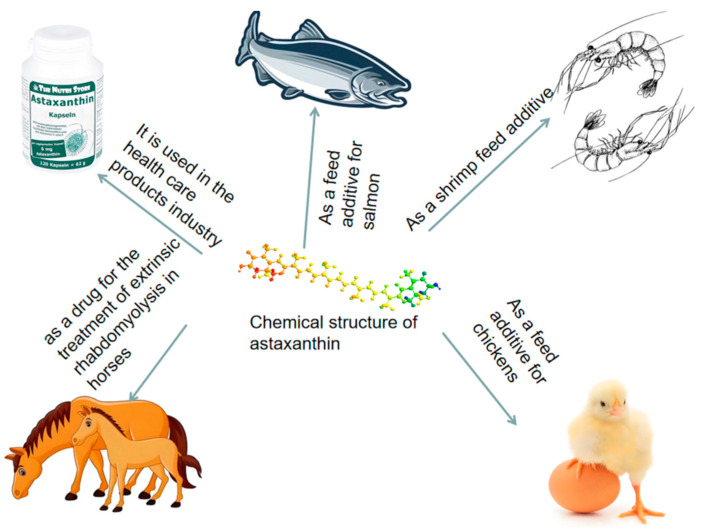
Chemical structure and applications of astaxanthin.

Astaxanthin is a keto-carotenoid with the chemical name 3,3′-dihydroxy-4,4′-diketone-β,β′-carotene (Figure 2). It appears as a red solid powder and is lipid-soluble, insoluble in water, and soluble in various organic solvents. The primary natural sources of astaxanthin include *Haematococcus pluvialis*, *Phaffia rhodozyma*, and crustacean byproducts [52,53,54]. Currently, astaxanthin-containing health products are widely available in the market.

The extraction methods for astaxanthin are diverse, including solvent extraction, acid extraction, oil-based methods, microwave-assisted extraction, and enzymatic methods. Studies have shown that hydrochloric acid extraction can achieve a recovery efficiency of up to 80%, while the oil-based method using olive oil can reach a maximum recovery efficiency of 93% [55].

#### 3.1.2. Functions and Mechanisms of Astaxanthin

**Figure 3:**
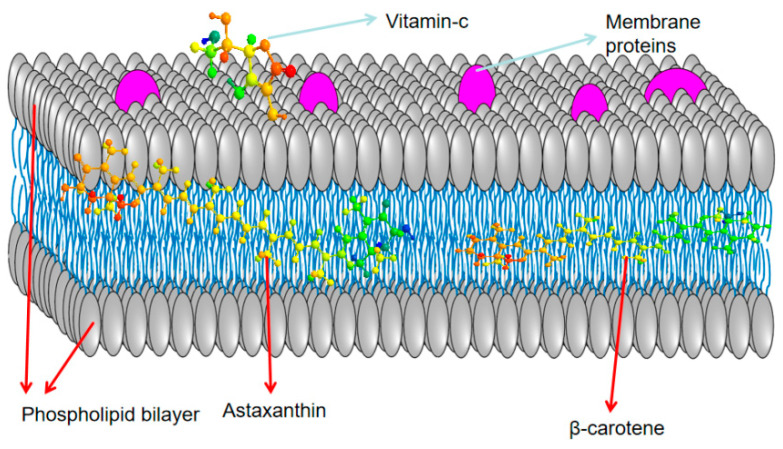
Superior position of astaxanthin in the cell membrane.

Consuming high concentrations of carotenoids can reduce the risk of chronic diseases such as cardiovascular diseases, cataracts, macular degeneration, and certain types of cancer [56]. Astaxanthin contains conjugated double bonds, hydroxyl groups, and keto groups, which confer potent antioxidative properties. These conjugated double bonds donate electrons and react with free radicals, converting them into more stable compounds and terminating the free radical chain reaction [57]. Astaxanthin can bind to cell membranes from the inside out (Figure 3), exhibiting excellent bioactivity [55].

In recent years, natural astaxanthin has gained significant attention in the nutritional supplement industry and is primarily marketed as a dietary supplement. Astaxanthin has demonstrated remarkable effectiveness in treating muscle-related disorders in mammals, such as exertional rhabdomyolysis in horses [58].

#### 3.1.3. Applications of Astaxanthin in Aquaculture

Astaxanthin plays a crucial role in pigment deposition in salmon farming [59]. In ornamental fish culture, its supplementation enhances the vibrancy of fish coloration [60]. Substantial evidence suggests that astaxanthin significantly impacts the reproductive performance of aquatic animals [61]. For instance, feeding *Litopenaeus vannamei* (whiteleg shrimp) with astaxanthin-enriched diets significantly increases hemocyte counts, phenoloxidase activity, and superoxide dismutase activity, thereby enhancing non-specific immune responses [62].

Additionally, astaxanthin-enriched feed improves the histological features and antioxidative capacity of the juvenile Indian white shrimp, *Fenneropenaeus indicus* [63]. Since aquatic animals cannot synthesize astaxanthin endogenously, they must obtain it from external sources. Consequently, astaxanthin is commonly incorporated into formulated feeds in aquaculture [64]. For large yellow croaker, *Larimichthys crocea*, astaxanthin supplementation significantly improves growth performance. Compared to a basal diet, feeding with 0.28–0.56 g of astaxanthin per kilogram of feed notably enhances blood health, antioxidative capacity, and immune responses [65].

The comprehensive review by Nakano and Wiegertjes [66] further elaborates on carotenoids, including astaxanthin’s multifunctional roles in aquaculture, including its potential applications as a natural feed additive to replace synthetic antioxidants. The review underscores astaxanthin’s benefits in enhancing product quality, supporting sustainable aquaculture practices, and addressing consumer demands for natural and safe aquaculture products.

In conclusion, astaxanthin represents a potent natural antioxidant with extensive applications in both health and aquaculture industries. Its multifaceted roles in mitigating oxidative stress, modulating signaling pathways, and improving aquaculture outcomes make it an indispensable component of modern aquatic farming strategies.

#### 3.1.4. Future Prospects of Astaxanthin in Aquaculture

As concerns over food safety grow and traditional medication use in aquaculture becomes more restricted, astaxanthin is increasingly regarded as a vital additive for its natural, non-toxic, and side-effect-free properties. However, the high production cost of astaxanthin at up to USD 718 per kilogram [67] limits its widespread application. Enhancing the efficiency of astaxanthin production and extraction will therefore be a key focus for future research.

Astaxanthin, as a natural pigment and antioxidant, also shows extensive potential in the food, pharmaceutical, and cosmetic industries. In the food industry, it serves as a natural colorant for candies and baked goods. In medicine, its robust antioxidative properties position it as a promising candidate for treating oxidative-stress-related diseases, such as cardiovascular conditions. Furthermore, astaxanthin has significant potential in the development of antioxidant cosmetics.

In conclusion, astaxanthin is expected to diversify and expand in application scope in the future. With deeper research and technological advancements, its utility and benefits in aquaculture are anticipated to grow further.

### 3.2. Polysaccharides: Antioxidative Functions and Practical Applications

Polysaccharides, one of the four fundamental biomolecules (proteins, nucleic acids, lipids, and carbohydrates), perform vital physiological functions in living organisms. They exhibit significant potential in various bioactivities, including anti-tumor, anti-inflammatory, antiviral, hypoglycemic, anti-aging, anticoagulant, and immune-enhancing effects [68]. Based on their sources, polysaccharides can be categorized into plant polysaccharides, animal polysaccharides, and bacterial polysaccharides (Table 2). Among these, plant polysaccharides stand out as a promising medicinal resource, playing crucial roles in managing oxidative-stress-related diseases ranging from inflammation to cancer. Both laboratory and clinical studies demonstrate that plant polysaccharides exhibit antioxidative, anti-inflammatory, cell-vitality-promoting, immune modulating, and anti-tumor properties [69]. Their wide availability, natural safety, and diverse biological activities make plant polysaccharides especially noteworthy. This section focuses on the functions, extraction methods, antioxidative mechanisms, and applications of plant polysaccharides in aquaculture.

#### 3.2.1. Functions and Extraction of Plant Polysaccharides

Plant polysaccharides are natural high-molecular-weight compounds formed by glycosidic bonds linking ten types of monosaccharides, such as mannose, glucose, and xylose [70]. They are widely present in various plant species such as blueberries and in Chinese herbal medicines such as astragalus, black plums, lemon peels, etc. Plant polysaccharides have no sweetness or reducing properties and are generally insoluble in cold water, soluble in hot water to form colloidal solutions, and insoluble in organic solvents such as ethanol. Numerous studies have confirmed the biological activities of plant polysaccharides, such as antioxidative activity [71], blood-glucose-lowering effects [72], and anti-tumor properties [73]. For example, *Astragalus membranaceus*, a perennial herb, exhibits immunomodulatory, hepatoprotective, diuretic, anti-aging, anti-stress, and anti-hypertensive effects [74]. Astragalus polysaccharides, as the main active component, also play a significant role in regulating gut microbiota composition and maintaining intestinal health [75]. Research has shown that the addition of Astragalus polysaccharides (APS) upregulates the differentiation of TNF α, IL-1 β, and other immune related genes in the gut of grass carp, while also increasing the proliferation of intestinal epithelial cells, thereby enhancing grass carp’s immune response and regulating the diversity and composition of grass carp gut microbiota [76]. Moreover, blueberries, known as the “king of berries”, are highly nutritious fruits rich in polysaccharides, anthocyanins, vitamins A and E, and SOD. Among these, polysaccharides are a key functional component and a subject of extensive research [77]. Blueberry polysaccharides neutralize excess free radicals in the body, reducing their damage to cells. At the same time, blueberry polysaccharides can promote the synthesis and secretion of antioxidant enzymes, enhance the body’s ability to clear free radicals, and thus play an antioxidant role.

With advancements in research technologies, plant polysaccharide extraction techniques have become more refined, enabling efficient extraction from various plant sources. Tian et al. [70] summarized extraction methods for astragalus polysaccharides, including solvent extraction, ultrasound-assisted extraction, microwave-assisted extraction, enzyme-assisted extraction, ultra-high-pressure extraction, and combined methods. Each method has its advantages and limitations. For instance, while water extraction is simple, it has low efficiency, with yields ranging from 3.57% to 14.76% after 45–90 min and a long processing time [78]. In contrast, ultra-high-pressure extraction achieves yields of 24.28% to 24.36% in just five minutes, albeit with high equipment requirements [79].

The potential of plant polysaccharide extraction extends to other fields. For instance, polysaccharides extracted from fermented bayberry pomace used in winemaking demonstrate excellent antioxidative properties, such as scavenging DPPH radicals, ABTS+ radicals, and reducing ferric ions [80]. Enzyme-assisted extraction of polysaccharides from ginseng fruits achieves a yield of 19.8 ± 0.01%, and the isolated α-pyran polysaccharides exhibit in vitro antioxidative activity, further highlighting their potential as natural antioxidants.

In summary, plant polysaccharides are functional substances with remarkable bioactivities and application value. By continuously optimizing extraction technologies, their utilization efficiency can be further improved, advancing their research and applications in aquaculture and other fields.

#### 3.2.2. Antioxidative Activity and Mechanisms of Plant Polysaccharides

Polysaccharides extracted from plants exhibit robust antioxidative properties characterized by multi-pathway, multi-target, and multi-effect mechanisms. Studies have shown that polysaccharides can regulate the expression of downstream antioxidative enzymes through endogenous oxidative stress pathways, such as the Nrf2-ARE pathway, to exert their antioxidative effects [81]. For example, astragalus polysaccharides (APS) improve cardiac function in rats by activating the Keap1-Nrf2/ARE signaling pathway to regulate antioxidative enzyme expression [82].

Another study found that selenium-enriched *Schisandra chinensis* polysaccharides (sSCP) significantly enhanced the viability of chicken embryo stem cells and the activities of antioxidative enzymes like SOD, CAT, and GSH-Px, while inhibiting apoptosis. The underlying mechanisms are likely related to the regulation of MAPK pathway protein expression and mitochondrial-dependent apoptosis signaling, thereby protecting cells from hydrogen-peroxide-induced oxidative damage [83].

Research on pumpkin polysaccharides has identified them as functional heteropolysaccharides composed of xylose, arabinose, glucose, rhamnose, galactose, and glucuronic acid [84]. Chemically modified phosphated pumpkin polysaccharides (PP1 and PP2) synthesized using phosphorus oxychloride-pyridine exhibited significant antioxidative capacities in superoxide anion scavenging experiments, with the low-substitution PP1 demonstrating particularly pronounced effects [85].

In addition to direct antioxidative effects, plant polysaccharides can enhance the immune system, indirectly improving the body’s antioxidative capacity. Gao et al.’s study showed that the derivative of pumpkin polysaccharide (PPe-s) prolongs the lifespan of *Caenorhabditis elegans* under hydrogen peroxide stress by upregulating the mRNA expression of daf-16, sod-1, sod-3, and skn-1; therefore, PPe-s can upregulate the expression levels of antioxidant-related genes and enhance the body’s antioxidant defense system [86]. By promoting immune function and cellular activity, polysaccharides offer great potential for applications in disease prevention and health maintenance, as well as being feed additives.

#### 3.2.3. Applications of Plant Polysaccharides as Food Additives in Aquaculture

As a natural food additive, plant polysaccharides are renewable, cost-effective, environmentally friendly, and highly efficient, aligning with modern food industry trends and consumer concerns about food safety and health. Significant progress has been made in their application to fish, shrimp, and crab farming. Research shows that plant polysaccharides can act as immune enhancers [87] and growth promoters [88], improving the immune capacity and growth performance of aquatic animals.

**Figure 4:**
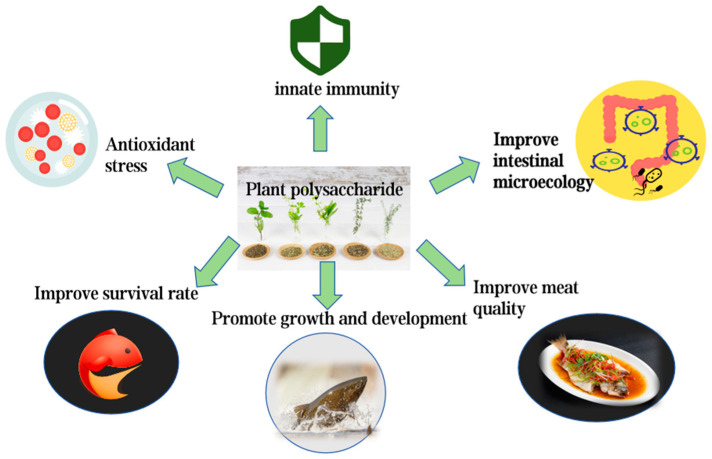
The effect of plant polysaccharides in aquatic products.

In aquaculture, plant polysaccharides are often used as feed additives (Figure 4). For instance, Chen et al. [89] demonstrated that adding 0.5% *Salvia miltiorrhiza* polysaccharides to the diet of juvenile hybrid sturgeon significantly enhanced antioxidative and non-specific immune capacities, improving survival rates and disease resistance against *Streptococcus iniae*.

Another study found that including ginger polysaccharides (GP) in the diet of crucian carp upregulated the expression of pro-inflammatory cytokines such as *TNF-α*, *IL-8*, *IFN-γ*, and *NF-κB*, while downregulating anti-inflammatory cytokines *IL-10* and *TGF-β*. This improved digestive enzyme activity, antioxidative capacity, and liver protection [90].

Additionally, plant polysaccharides can significantly improve the gut microbiota of aquatic animals. By promoting beneficial bacteria growth and suppressing harmful bacteria, they enhance intestinal barrier protection. For example, supplementing feed with goji berry polysaccharides (*Lycium Barbarum* polysaccharides, LBP) improved the gut microbiota composition of *Luciobarbus capito*, significantly increasing liver antioxidative enzyme, intestinal digestive enzyme, and liver immune enzyme activities, thereby maintaining gut health [91].

Pomelo is a citrus fruit with great potential for use as a drug or feed additive in aquaculture. The crude polysaccharide extracted and purified from pomelo has a good protective effect on cell apoptosis mediated by oxidative stress, inflammation, and mitochondrial dysfunction. In the study of Liu et al. [92], they found that feeding different doses of pomelo polysaccharide (YZW-A) to *Epinephelus coioides* reduced H_2_O_2_ induced oxidative stress at doses of 100 μg/mL and 200 μg/mL, and it reduced gene expression of pro-inflammatory cytokines (TNF-α, IL-β, and IL-6) regulated by NF-κB. Due to the reduction of oxidative stress, mitochondrial-dysfunction-mediated cell apoptosis was correspondingly alleviated.

In summary, the application of plant polysaccharides in aquaculture not only improves the health and productivity of aquatic animals but also provides effective solutions for promoting green aquaculture and sustainable development.

Currently, research on plant polysaccharides in aquaculture primarily focuses on their effects on the growth performance, immunity, and intestinal digestion of aquatic organisms [93,94,95]. However, studies on their impact on muscle quality and sensory attributes, such as taste and texture, of aquatic products are relatively limited. Expanding research in this area could provide a more comprehensive evaluation of the potential of plant polysaccharides in improving the overall quality of aquatic products.

Despite the promising applications of plant polysaccharides in aquaculture, their practical use remains in the preliminary stages of investigation. The existing studies require further exploration and refinement regarding their application effects and underlying mechanisms. Additionally, significant differences in the responses of various aquatic species to plant polysaccharides highlight the need for systematic research on appropriate dosage levels for specific aquaculture species, providing a scientific basis for practical farming applications.

To further enhance the application efficiency of plant polysaccharides, research could explore their combination with other natural antioxidants. By optimizing composite formulations, it would be possible not only to reduce overall costs but also to more effectively enhance the antioxidative capacity, immune functions, and production performance of aquatic animals, thereby promoting the sustainable development of green aquaculture.

### 3.3. The Vitamin Family: Oxidative Stress Mitigation and Nutritional Applications

#### 3.3.1. Vitamins and Their Critical Roles in Nutrients and Health

Vitamins are trace organic compounds essential for maintaining normal physiological functions in humans and animals, and the term is a specialized concept in nutrition. Most vitamins cannot be synthesized by organisms and must be obtained through dietary intake, although a few can be synthesized within the body or produced by gut microbiota [96]. Vitamins play irreplaceable roles in growth, metabolism, and development, and they are classified as one of the six essential nutrients for humans (carbohydrates, fats, proteins, minerals, vitamins, and water). To date, at least 30 different compounds are classified as “vitamins”, with more than 20 known to be critical for biological health [97]. The following table introduces the characteristics and sources of common types of vitamins (Table 3).

#### 3.3.2. Mechanisms of Action of Different Vitamins

Vitamins are classified into two major categories based on their solubility: fat-soluble vitamins (A, D, E, and K) and water-soluble vitamins (B-complex and C) [98]. Water soluble vitamins contain polar groups and are water-soluble, making them difficult to store in the body. Fat soluble vitamins contain non-polar functional groups, are lipophilic and soluble in fats and organic solvents, and can be stored in the body. Their unique biochemical properties determine the differences in their absorption, transportation, metabolism, and mode of action, thus playing different roles in the human body. Vitamins can exert their biological functions in various ways, among which activating gene expression is an important mode of action. For example are the genomic and non-genomic roles of vitamin D, in which vitamin D activates gene expression by binding to vitamin D receptors, as well as its important roles and related signaling pathways in calcium and phosphorus metabolism, bone health, immune function, and other areas [99]. As another example, vitamin E activates or inhibits the expression of specific genes by regulating the activity of transcription factors, affecting intracellular signaling pathways and other mechanisms in physiological processes such as antioxidant defense, cell proliferation, and immune regulation [100]. Their mechanisms of action in the body include the following aspects:

Participation in Enzymatic Reactions as Coenzymes or Cofactors

Many vitamins are indispensable coenzymes or cofactors in enzymatic reactions. For instance, various members of the B-complex vitamins (e.g., B1, B6, and B12) serve as coenzymes in the metabolism of carbohydrates, proteins, and lipids, contributing to energy conversion and biosynthesis [101].

Antioxidant Effects

**Figure 5:**
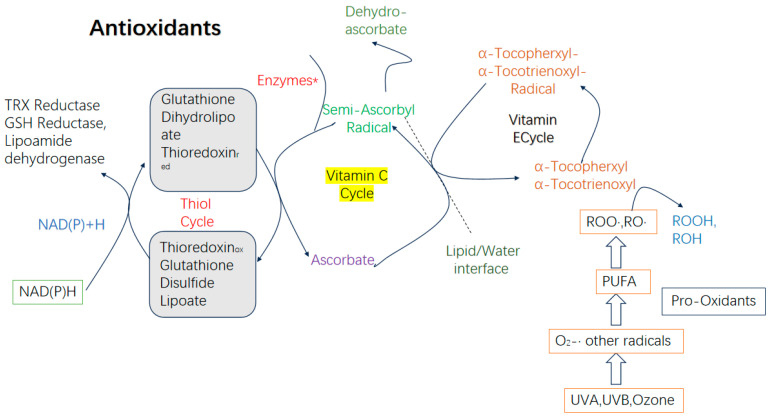
The antioxidant application of vitamins.

Vitamin C is a potent antioxidant that scavenges free radicals in the body, protecting cells from oxidative damage. Additionally, it reduces oxidized vitamin E to its active form, allowing it to continue its antioxidative role. Vitamin E, as a vital fat-soluble antioxidant, effectively protects biological membranes from free radical attacks. By binding with free radicals, it forms stable compounds, thereby interrupting the chain reaction of free radical damage (Figure 5) [102,103].

Maintenance of Cellular Structure and Function

Vitamins play a critical role in maintaining cellular structure and function. For example, vitamin A is pivotal in cell differentiation and the visual system, while vitamin D is indispensable for maintaining calcium–phosphorus metabolic balance [104].

Regulation of Gene Expression

Certain vitamins directly regulate gene expression by binding to nuclear receptors, thereby influencing cellular metabolism and growth. For instance, vitamin D binds to its receptor VDR to regulate a range of genes associated with bone health and immune function [105].

Immune Modulation

Vitamins are also key players in immune regulation. For example, vitamins A and D enhance immune cell functions and regulate the secretion of inflammatory cytokines, thereby promoting a healthy immune system [106].

#### 3.3.3. Application of Vitamin Antioxidative Properties in Aquaculture

The antioxidative applications of vitamins in aquaculture primarily stem from their ability to neutralize free radicals. Free radicals, harmful byproducts of metabolic processes, can damage cells and negatively impact the growth, development, and health of aquatic animals.

For example, studies have demonstrated that adding appropriate levels of vitamin E to fish feed improves the growth rate of carp, enhances their antioxidative capacity, and mitigates oxidative stress damage [107]. Similarly, vitamin C plays a vital role in the physiological processes and health of aquatic animals. Recent research has explored the effects of vitamin C on growth, antioxidative status, immunity, disease prevention, reproductive performance, and the interactions between dietary vitamin C and gut microbiota in farmed aquatic species [108]. One study examined the impact of varying dietary levels of vitamin C on the growth performance, muscle composition, antioxidative status, and enzyme activities of freshwater prawns (*Macrobrachium malcolmsonii*), highlighting its beneficial effects [109].

These findings indicate that the antioxidative properties of vitamins have broad applications in aquaculture. Vitamins not only enhance the immunity of aquatic animals but also alleviate environmental stress and promote growth and development. Future research should focus on understanding the synergistic mechanisms of vitamins in aquaculture, developing innovative formulations and application methods, as well as improving the quality of aquatic products while minimizing environmental impact.

### 3.4. Polyphenols: Molecular Mechanisms and Aquaculture Benefits

#### 3.4.1. Introduction to Polyphenols

Polyphenolic compounds are widely found in natural plants such as grapes, apples, and blueberries. Grapes, in particular, have skins and seeds rich in polyphenolic compounds, including proanthocyanidins and resveratrol. The main components of polyphenolic compounds are shown in the following figure (Table 4).

**Figure 6:**
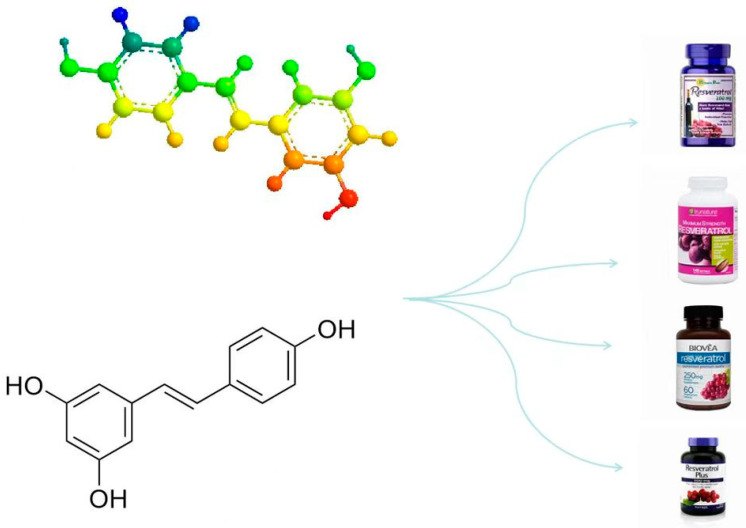
Structure of trans-resveratrol (left) and its applications in the healthcare industry.

Resveratrol, a natural antioxidant, was first extracted from *Veratrum* species (family *Liliaceae*) by Michio Takaoka [110]. It was later identified in studies on plants such as grapes and peanuts [111]. Resveratrol is synthesized in plants when exposed to stress and offers numerous benefits, including antioxidative, anti-aging, vascular protective, and neuroprotective effects [112]. Compared to synthetic antioxidants, resveratrol has several advantages, such as lower environmental impact during extraction and remarkable effectiveness, making it a promising natural antioxidant. The molecular formula of resveratrol is C_14_H_12_O_3_, with the scientific name 3,4,5-trihydroxy-1,2-diphenylethylene. In its pure form, it appears as white needle-shaped crystals that are poorly soluble in water but readily soluble in organic solvents. Resveratrol exists in two isomeric forms: cis- and trans-resveratrol. Although both structures exhibit biological activity, studies suggest that trans-resveratrol, due to its higher stability and bioactivity, is more advantageous for practical applications (Figure 6) [113].

#### 3.4.2. Extraction Methods of Resveratrol

The extraction of resveratrol leverages its physicochemical properties and includes methods such as microwave-assisted separation, macroporous resin adsorption, solvent extraction, and supercritical fluid extraction. Each method has distinct advantages and limitations:

Microwave-Assisted Separation: Efficient, energy-saving, and yields high-purity extracts; however, its high-temperature effects can be detrimental to thermosensitive substances.

Solvent Extraction: Widely used due to its simplicity and broad applicability, but it often suffers from low extraction efficiency and high costs.

Supercritical Fluid Extraction: High efficiency, excellent product quality, and environmentally friendly; however, its high production cost limits large-scale applications.

After crude extraction, purification is necessary to obtain pure trans-resveratrol. Among various methods, high-performance counter-current chromatography (HPCCC) has demonstrated superior performance in purification, making it an ideal choice for isolating trans-resveratrol [114]. Continuous optimization of extraction and purification processes could enhance the efficiency and application potential of resveratrol, providing feasible technical support for further development.

#### 3.4.3. Antioxidative Properties of Resveratrol

Resveratrol is a polyphenolic compound containing three hydroxyl groups in its structure, enabling it to bind with free radicals in the body. This binding neutralizes free radicals by eliminating excess electrons, preventing further cellular damage and exerting antioxidative effects [115]. Additionally, resveratrol enhances the body’s antioxidative capacity by activating the activities of antioxidative enzymes [116].

**Figure 7:**
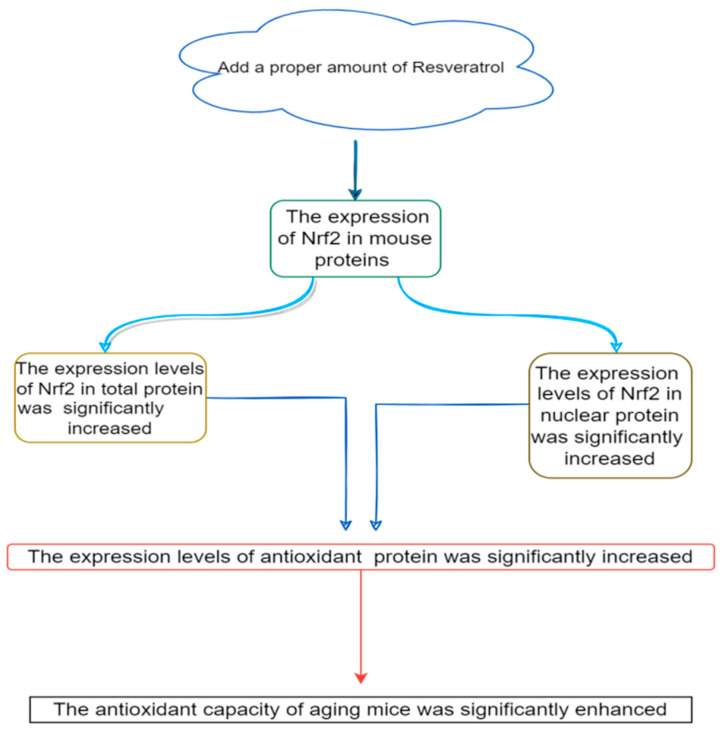
Mechanism of antioxidant activity of resveratrol by regulating the Nrf2 pathway.

Resveratrol’s antioxidative mechanisms also involve the regulation of signaling pathways. By activating the Nrf2 signaling pathway and the phosphatidylinositol 3-kinase/protein kinase B (PI3K/Akt) signaling pathway, resveratrol promotes the expression of antioxidative genes, thereby amplifying its antioxidative efficacy (Figure 7).

For instance, in a study on rats with myocardial ischemia/reperfusion injury, intraperitoneal injection of 20 mg/kg resveratrol significantly increased the expression levels of PI3K and phosphorylated Akt (p-Akt) in myocardial tissue. It also reduced malondialdehyde (MDA) levels in plasma and myocardium while enhancing the activity of SOD [117]. These findings indicate that resveratrol not only directly scavenges free radicals but also indirectly strengthens the body’s antioxidative capacity through signaling pathway regulation.

#### 3.4.4. Application of Resveratrol as a Feed Additive

Related papers have pointed out that a high-fat diet can reduce the antioxidant capacity of carp, triggering inflammatory reactions and lipid deposition. Resveratrol can significantly increase the final body weight, weight gain rate, and fatness of carp; reduce feed conversion rate; and significantly increase the activities of total superoxide dismutase, catalase, and glutathione peroxidase in carp serum through the Nrf2 signaling pathway, as well as reduce malondialdehyde content and upregulate the expression of genes such as catalase, copper zinc superoxide dismutase, and glutathione peroxidase 1a and 1b in the liver, thereby enhancing carp’s antioxidant capacity [118]. In another study, researchers divided grass carp into multiple groups and added different concentrations of resveratrol. After feeding for a period of time, various indicators of grass carp were tested. It was found that adding resveratrol to the feed significantly increased the activity of antioxidant enzymes in grass carp, such as superoxide dismutase, catalase, and glutathione peroxidase. These enzymes can effectively eliminate excessive free radicals in the body, reduce oxidative damage, and enhance the antioxidant capacity of grass carp [119].

Studies have shown that incorporating resveratrol into feed significantly enhances the antioxidative capacity of farmed animals. For instance, supplementing resveratrol in the diet of snakehead fish (*Channa argus*) effectively improves their antioxidative capabilities [120]. Similarly, experiments with rainbow trout demonstrated that resveratrol supplementation not only enhances antioxidative capacity but also significantly improves feed utilization efficiency [121]. In other model studies, Hou et al. found that resveratrol, by activating the PI3K/Akt/mTOR signaling pathway, improves neurological function in ischemic rats; reduces neuronal damage; and significantly upregulates the expression of proteins such as p-Akt, p-mTOR, and B-cell lymphoma-2 (Bcl-2) [122]. These findings suggest that resveratrol achieves its antioxidative effects through the regulation of specific signaling pathways.

Moreover, oxidative stress can activate Toll-like receptor (TLR) signaling pathways, promoting the release of pro-inflammatory cytokines and triggering inflammatory responses. Resveratrol counteracts this by inhibiting TLR signaling transduction, thus exerting both antioxidative and anti-inflammatory effects. Upon binding with ligands, TLRs dimerize and transmit signals through the TLR domain, initiating downstream responses [122]. These results indicate that resveratrol’s antioxidative properties are mediated through multiple pathways.

In summary, adding resveratrol to feed not only significantly enhances the antioxidative capacity of aquatic animals but also supports its antioxidative and anti-inflammatory effects by regulating multiple signaling pathways. These findings provide a scientific foundation and valuable insights for the rational application of resveratrol in the future.

### 3.5. Flavonoids: Multifunctional Antioxidants in Health and Aquaculture

#### 3.5.1. Extraction and Functions of Flavonoids

Flavonoids are secondary metabolites widely found in plants, including fruits, vegetables, tea, and wine [123]. With a flavone (2-phenylchromone) backbone, flavonoids are characterized as pigments with yellow or other colors. Their basic structure consists of three rings: two benzene rings (rings A and B) connected by a three-carbon chain (ring C), forming the typical C6-C3-C6 skeleton.

Flavonoids can be categorized into several subclasses based on their molecular structure, including flavonols, flavones, isoflavones, anthocyanins, flavanones, flavanols, and chalcones. These compounds perform various biological functions in plants, such as acting as antioxidants, antimicrobial agents, or signaling molecules [123]. Additionally, flavonoids play a critical role in plants’ responses to environmental stress, supporting growth and adaptability [124].

In recent years, researchers have developed various extraction methods to enhance the efficiency and purity of flavonoid extraction. For instance, ultrasonic-assisted extraction (UAE) has proven effective for extracting antioxidant compounds, including flavonoids, from *Aloysia citrodora L.* (lemon verbena) [125]. Here are some common extraction methods and sources of flavonoids (Table 5).

#### 3.5.2. Antioxidative Activity and Mechanisms of Flavonoids

**Figure 8:**
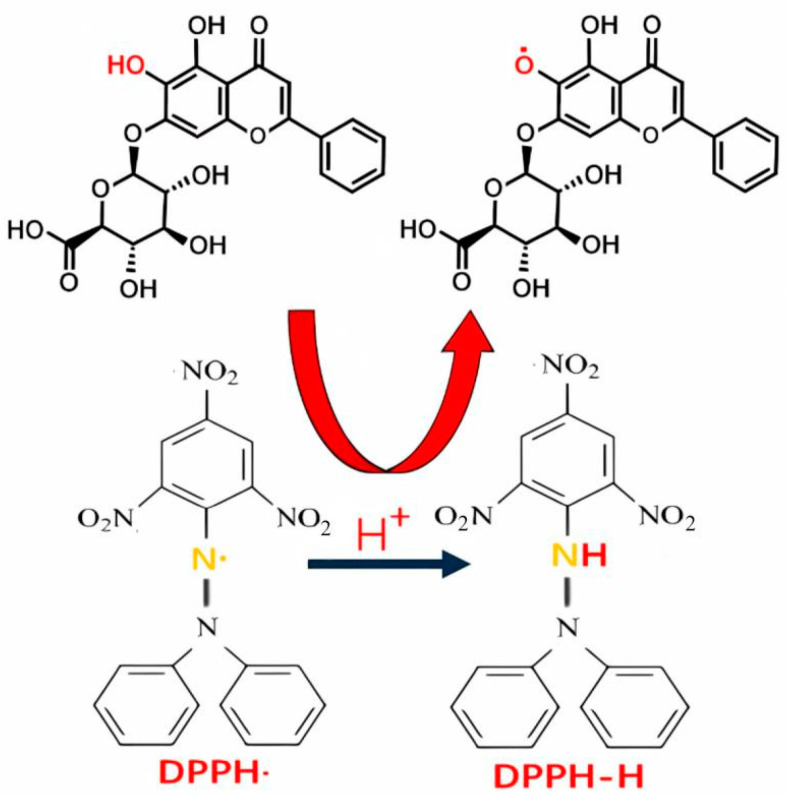
The mechanism of using baicalin to scavenge DPPH radicals.

Flavonoids possess significant antioxidative activity due to their unique structure, which enables them to effectively scavenge free radicals and superoxide anions, thereby mitigating oxidative damage. For example, the mechanism of flavonoids scavenging DPPH free radicals has been well-documented. Flavonoids, such as baicalin, neutralize free radicals by donating hydrogen atoms or electrons, thus terminating chain reactions (Figure 8). Additionally, flavonoids inhibit oxidative reactions through metal ion chelation. For instance, quercetin has demonstrated the ability to chelate metal ions, effectively suppressing the formation of advanced glycation end products (AGEs) and thereby reducing oxidative damage [126]. Simultaneously, flavonoids can induce the increased expression and activity of intracellular antioxidant enzymes, such as catalase. These antioxidant enzymes can catalyze the decomposition of reactive oxygen species (ROS), thereby enhancing the cell’s own antioxidant defense capabilities [127]. These properties highlight the potential of flavonoids as powerful natural antioxidants with broad applications in health and industry.

#### 3.5.3. Specific Applications of Flavonoids

Flavonoids, due to their antioxidative properties, demonstrate vast potential in various aspects of aquaculture. For instance, adding flavonoids (e.g., catechins from rosemary extract) to fish oil systems significantly reduces lipid oxidation levels, thereby enhancing the stability of fish oil. This not only prolongs the shelf life of fish oil but also preserves its nutritional value [128]. Similarly, incorporating flavonoids (e.g., catechins from green tea extract) into aquaculture feed has shown effective inhibition of oxidative reactions. In a study on olive flounder feed, flavonoids markedly reduced oxidation in both stored feed and fish tissues, improving product freshness and nutritional quality [129]. Moreover, flavonoids can inhibit some enzymes that generate ROS, such as lipoxygenase, cyclooxygenase, and xanthine oxidase, and this inhibitory effect helps reduce the production of ROS in zebrafish [130].

#### 3.5.4. Future Prospects and Challenges of Flavonoids

With the rapid development of aquaculture and the growing demand for sustainable production, flavonoids are emerging as vital resources for enhancing aquaculture efficiency and product quality. As natural bioactive compounds, flavonoids have the potential to replace some synthetic antioxidants and offer new solutions for aquaculture by improving feed antioxidative properties and nutritional value.

However, the widespread application of flavonoids faces several challenges, such as limited stability in feed, low bioavailability, and variability in effectiveness across different aquatic species.

Stability Issues: Flavonoids are prone to degradation under high temperatures or prolonged storage, which can diminish their antioxidative efficacy.

Bioavailability Concerns: Significant differences in the absorption and metabolism of flavonoids among aquatic species limit their scalability for large-scale farming applications.

Future research should focus on improving the stability and bioavailability of flavonoids while optimizing their application strategies for various aquaculture species. Exploring the synergistic effects of flavonoids with other natural antioxidants and developing innovative feed formulations will further expand their applications in aquaculture, supporting the sustainable development of the industry.

### 3.6. Analytical Techniques for Antioxidants: Advanced Methods and Applications

Antioxidant analysis plays a pivotal role in understanding and characterizing the biochemical properties of natural antioxidants. Advanced methodologies are essential to accurately evaluate antioxidant activity, elucidate their mechanisms of action, and provide reliable data to support broader applications. This section introduces state-of-the-art analytical techniques specifically designed to address the inherent complexity of antioxidants, showcasing their relevance and importance in advancing antioxidant research.

(1). High-Performance Liquid Chromatography (HPLC) and LC-MS/MS

High-performance liquid chromatography (HPLC) coupled with diode array detection (DAD) and mass spectrometry (MS) is a cornerstone for identifying and quantifying specific antioxidants such as polyphenols, flavonoids, and carotenoids [131,132]. These methods offer exceptional sensitivity and specificity. Sample extraction involves methanol–water solutions, with chromatographic separation achieved on a C18 column using a gradient elution system (acetonitrile and water with 0.1% formic acid). Detection wavelengths are optimized at 280 nm for polyphenols and 450 nm for carotenoids.

(2). Electron Paramagnetic Resonance (EPR) Spectroscopy

EPR spectroscopy is a cutting-edge tool for directly quantifying free radicals and evaluating antioxidant capacities. Utilizing spin-trapping agents, this technique enables real-time measurement of radical concentrations, providing direct insights into antioxidant efficacy [133].

(3). Enzymatic Antioxidant Activities

Superoxide Dismutase (SOD): Measured using a xanthine-oxidase-based method that tracks the reduction of nitroblue tetrazolium (NBT) at 560 nm [134].

Catalase (CAT): Hydrogen peroxide decomposition is monitored spectrophotometrically at 240 nm [135].

Glutathione Peroxidase (GPx): A coupled assay with glutathione reductase quantifies the consumption of NADPH at 340 nm [136].

(4). Non-Enzymatic Antioxidants

Reduced Glutathione (GSH): Quantified using Ellman’s reagent [137].

Total Antioxidant Capacity (TAC): Evaluated through the ferric reducing ability of plasma (FRAP) and Trolox equivalent antioxidant capacity (TEAC) assays [138].

(5). Oxidative Stress Biomarkers

Malondialdehyde (MDA): A marker of lipid peroxidation, MDA levels are quantified using HPLC with fluorescence detection [139].

Protein Carbonyls and 8-Hydroxy-2′-Deoxyguanosine (8-OHdG): These biomarkers of protein and DNA oxidative damage are assessed via GC-MS and ELISA-based assays [140].

These advanced techniques provide unparalleled accuracy and sensitivity in evaluating antioxidant activity, enabling a comprehensive understanding of their mechanisms and applications. By leveraging these methods, researchers can precisely characterize antioxidant properties, correlate them with biological outcomes, and enhance the applicability of natural antioxidants in aquaculture systems.

## 4. Future Prospects of Natural Antioxidants in Aquatic-Product-Related Fields

Natural antioxidants, known for their excellent antioxidative and antimicrobial properties, demonstrate vast potential in aquatic-product-related fields, particularly in feed additives and seafood preservation. When incorporated into animal feed, natural antioxidants effectively protect against lipid oxidation, protein peroxidation, and microbial contamination, thereby improving feed quality and safety while extending shelf life. For example, adding phenolic compounds to fish feed not only enhances feed palatability but also significantly increases feed intake and production performance in farmed animals [141].

Seafood, being nutrient-rich and high in water content, is highly susceptible to spoilage caused by microbial invasion and chemical reactions. Studies confirm that natural antioxidants, such as polyphenolic compounds, can inhibit oxidative processes and delay microbial growth in seafood. This not only extends shelf life but also preserves the original nutritional value of the products [142].

Furthermore, natural antioxidants play a vital role in enhancing seafood safety. Essential oils (EOs) derived from Mediterranean medicinal herbs, such as rosemary (*Rosmarinus officinalis*), thyme (*Origanum vulgare subsp. hirtum*), and savory (*Satureja thymbra*), contain potent antioxidative components like cinnamic alcohol, p-cymene, and γ-terpinene. These EOs exhibit both strong antimicrobial and antioxidative activities [143,144].

With the discovery of new natural antioxidants, such as *Filipendula ulmaria* tinctures [145,146], the diversity of these compounds has expanded, offering more options for seafood preservation. To enhance the extraction efficiency and application efficacy of natural antioxidants, advanced technologies like nanotechnology and biotechnology can be utilized to improve their stability and bioavailability [147,148].

Through interdisciplinary research and technological innovations, natural antioxidants are poised to play a more significant role in aquaculture and seafood processing, promoting sustainable development while meeting consumer demand for high-quality seafood.

## 5. Conclusions

Natural antioxidants, including carotenoids, polysaccharides, vitamins, polyphenols, and flavonoids, play a vital role in mitigating oxidative stress and enhancing the health and productivity of aquatic species. This review consolidates existing knowledge on their antioxidative mechanisms, practical applications, and benefits in aquaculture systems. The findings underscore the ability of these compounds to neutralize reactive oxygen species (ROS), protect cellular components from oxidative damage, and improve immune responses and stress tolerance in fish and other aquatic organisms. For industrial applications, the incorporation of natural antioxidants into aquafeeds offers a promising strategy to enhance growth performance, disease resistance, and product quality, while aligning with sustainable and eco-friendly aquaculture practices. However, their effectiveness depends on factors such as dosage, bioavailability, and the specific needs of the target species. Practical applications require optimizing feed formulations to achieve synergistic effects, conducting species-specific trials to determine ideal concentrations and delivery methods, and developing cost-effective extraction and production methods to ensure scalability in commercial aquaculture. Future research should focus on elucidating the precise molecular mechanisms of these compounds, their interactions with other feed additives, and their long-term effects on aquatic health and environmental sustainability. By addressing these areas, natural antioxidants can be better integrated into aquaculture practices, contributing to the industry’s growth and resilience. This review provides a framework for researchers and industry stakeholders to leverage the potential of natural antioxidants, offering actionable insights for advancing sustainable aquaculture systems.

## Figures and Tables

**Table 1:** Common carotenoids and their extraction techniques and sources.

Name	Characteristic	Extraction Technique	Source
β-Carotene	Antioxidant and eye protection	Organic solvent extraction	carrots, pumpkin
Lycopene	Antioxidant and prevents cardiovascular disease	Distillation concentration	tomato
Lutein	Antioxidant and eye protection	Supercritical fluid extraction	collard
Astaxanthin	Antioxidant, anti-inflammatory	Ultrasound-assisted extraction	Haematococcus pluvialis
Zeaxanthin	Antioxidant and eye protection	Enzymatic digestion	corn

**Table 2:** Types of polysaccharide natural antioxidants and their properties, extraction methods, and sources.

Type	Category	Characteristic	Extraction Method	Source
Plant polysaccharides	Natural polymer compounds	Water soluble, viscous, enhances immunity, anti-tumor, lowers blood sugar	Hot water extraction method, alkaline extraction method	Chinese herbal medicines (wolfberry, astragalus), grains (oats, corn), fruits and vegetables (kelp, pumpkin)
Animal polysaccharides	Bioactive polysaccharides	Lubricate joints, moisturize skin, promote cell repair	Acid alkali extraction method, organic solvent extraction method	Animal tissues (cartilage, skin, mesentery), body fluids (plasma, urine)
Bacterial polysaccharide	Microbial polysaccharide	Produced by bacterial fermentation. Enhance immunity, anti-tumor, as a vaccine adjuvant, etc.	Fermentation extraction method, organic solvent precipitation method	Bacterial fermentation broth (lactic acid bacteria, yeast, actinomycetes)

**Table 3:** Characteristics and sources of different types of vitamins.

Vitamin Type	Characteristic	Function	Extraction Method	Source
Vitamin A	Fat-soluble	Maintains vision, skin health, and normal function of epithelial tissues	Extraction with organic solvents	Animal livers, dairy products, eggs, vegetables and fruits rich in carotenoids (such as carrots, where carotenoids can be converted into vitamin A)
Vitamin B1 (thiamine)	Water-soluble	Involved in carbohydrate metabolism and has effects on the nervous system, etc.	Obtained through separation and purification methods	Yeast, brown rice, whole wheat, beans, lean meat, etc.
Vitamin B2 (riboflavin)	Water-soluble	Involved in biological oxidation processes in the body and related to mucosal health	Often extracted from microbial fermentation products or animal and plant materials rich in it	Dairy products, eggs, meat, cereals, vegetables, etc.
Vitamin C (ascorbic acid)	Water-soluble	Antioxidant, promotes collagen synthesis, enhances immunity, etc.	Extracted through processes such as pressing, filtering, concentrating, and crystallizing fruits and vegetables	Fresh fruits (oranges, lemons), vegetables (broccoli, green peppers)
Vitamin D	Fat-soluble	Promotes calcium absorption and bone development	Extracted from fish oil, etc., and partially synthesized through ultraviolet radiation on related substances	Marine fish, animal livers, eggs, and also synthesized partially by human skin through ultraviolet radiation
Vitamin E	Fat-soluble	Antioxidant, protects cells from damage caused by free radicals	Extracted from vegetable oils, nuts, and other raw materials using extraction and other methods	Vegetable oils (corn oil, olive oil), nuts, leafy vegetables

**Table 4:** Types, sources, functions, extraction methods, and characteristics of polyphenolic compounds.

Name	Function	Source	Extraction Method	Characteristic
Tea polyphenols	Antioxidant, antibacterial, lipid-lowering, cardiovascular protection	Tea leaves, such as green tea, black tea, oolong tea, etc.	Hot water extraction	Astringent taste, water-soluble and fat-soluble, affected by heat and light
Anthocyanins	Antioxidant, anti-inflammatory, eye protection, giving vivid colors to plants	Blueberries, purple potatoes, black goji berries, cranberries, etc.	Extraction with acidic ethanol solution	Water-soluble; sensitive to light, heat, and oxygen; should be stored at low temperature, away from light and oxygen
Resveratrol	Antioxidant, anticancer, anti-aging	Grapes, peanuts, Polygonum cuspidatum, etc.	Ultrasound-assisted extraction	White needle-like crystals, insoluble in water, easily soluble in organic solvents
Ellagic acid	Antioxidant, anticancer, antibacterial	Pomegranates, strawberries, walnuts, raspberries, etc.	Extraction with organic solvent reflux	Yellow needle-like crystals, slightly soluble in water, soluble in organic solvents, strongly acidic

**Table 5:** Types, sources, functions, extraction methods, and characteristics of flavonoids.

Name	Characteristics	Extraction Methods	Source
Quercetin	Antioxidant, anti-inflammatory, antibacterial	Supercritical fluid extraction technology	Fruits, vegetables, tea, etc.
Anthocyanidins	Antioxidant, anti-inflammatory	Ultrasound-assisted extraction technology	Fruits, berries
Isoflavones	Antioxidant, antitumor	Deep eutectic solvent extraction technology	Soybeans, legumes
Catechins	Antioxidant, antibacterial	Supercritical CO_2_ extraction technology	Green tea, tea
Flavones	Antioxidant, anti-inflammatory	Solvent-mediated extraction technology	Flowers, spices
Flanones	Antioxidant, antiviral	Ultrasound-assisted extraction technology	Citrus fruits
Chalcones	Antioxidant, anti-inflammatory	Deep eutectic solvent extraction technology	Flowers, spices

## Data Availability

No new data were created or analyzed in this study. Data sharing is not applicable to this article.
